# Supplementary material for: Forced Overexpression and Knockout Analysis of SLC30A and SLC39A Family Genes Suggests Their Involvement in Establishing Resistance to Cisplatin in Human Cancer Cells
Source: Int J Mol Sci. 2024 Nov 9;25(22):12049. doi: 10.3390/ijms252212049 (PMC11594112; doi:10.3390/ijms252212049)
Supplement: Supplementary file 1 [file ijms-25-12049-s001.zip › Supplementary Figure S1.docx]

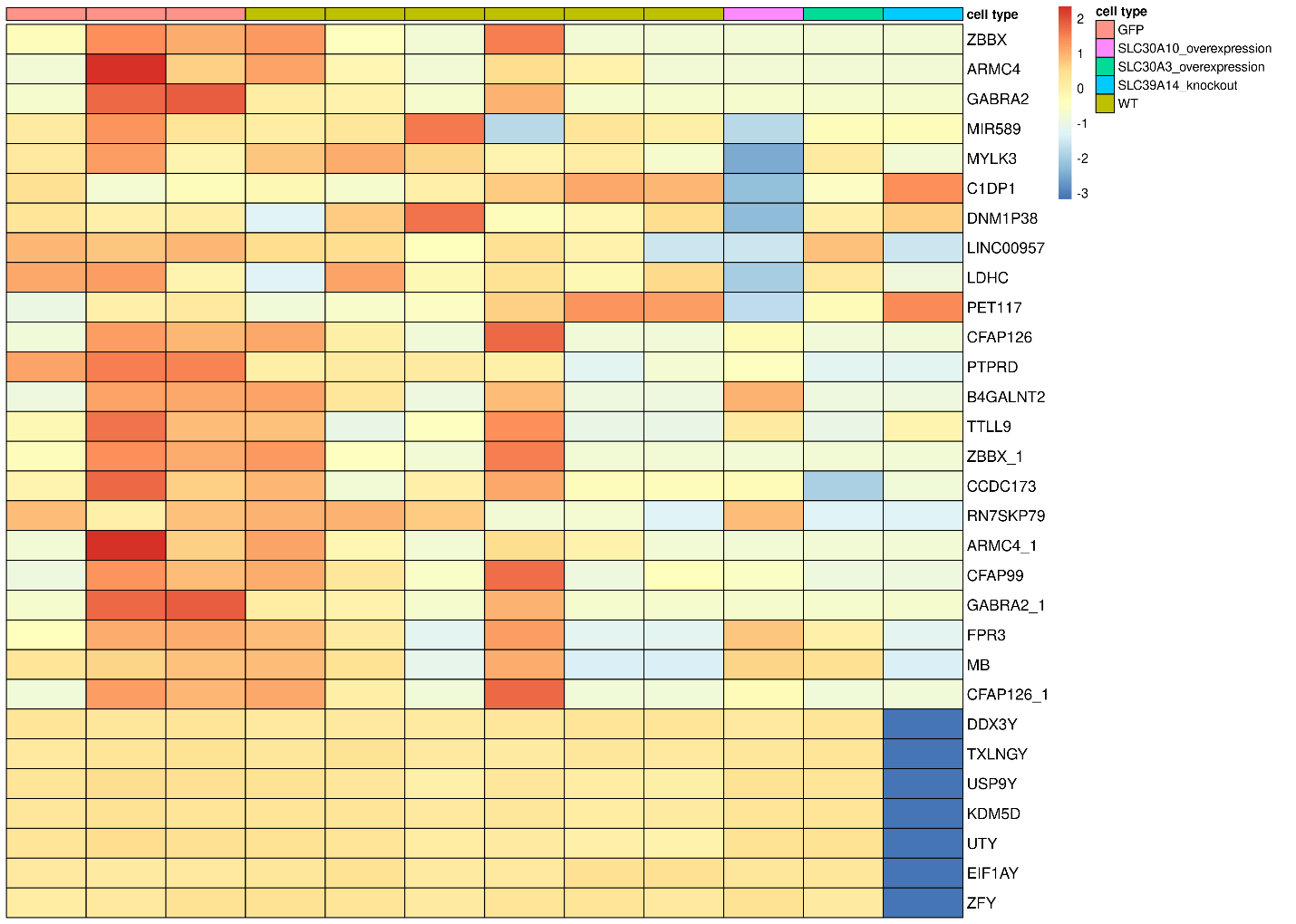


**Supplementary Figure S1**. **A) HCT-15 top downregulated genes.** From left to right, the figure displays control groups followed by the analyzed groups. Genes are arranged vertically in descending order of log2 fold change (log2FC), presenting the top 10 genes for each analyzed group, excluding controls. For the 'down' category, the genes represent the lowest log2_FC values. The values within the cells correspond to z-scores.


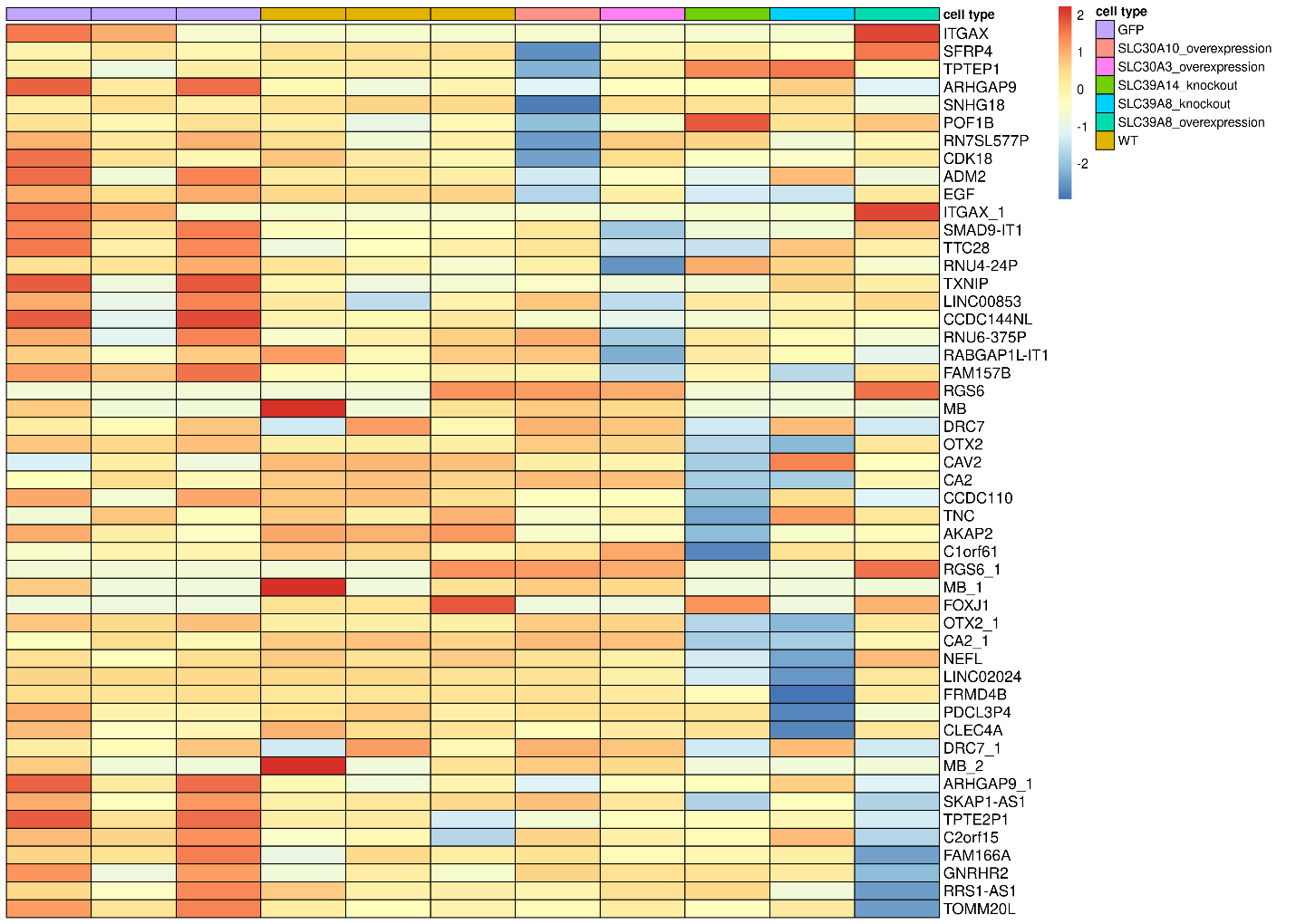


**Supplementary Figure S1. B) HuTu80 top downregulated genes.** From left to right, the figure displays control groups followed by the analyzed groups. Genes are arranged vertically in descending order of log2 fold change (log2FC), presenting the top 10 genes for each analyzed group, excluding controls. For the 'down' category, the genes represent the lowest log2_FC values. The values within the cells correspond to z-scores.


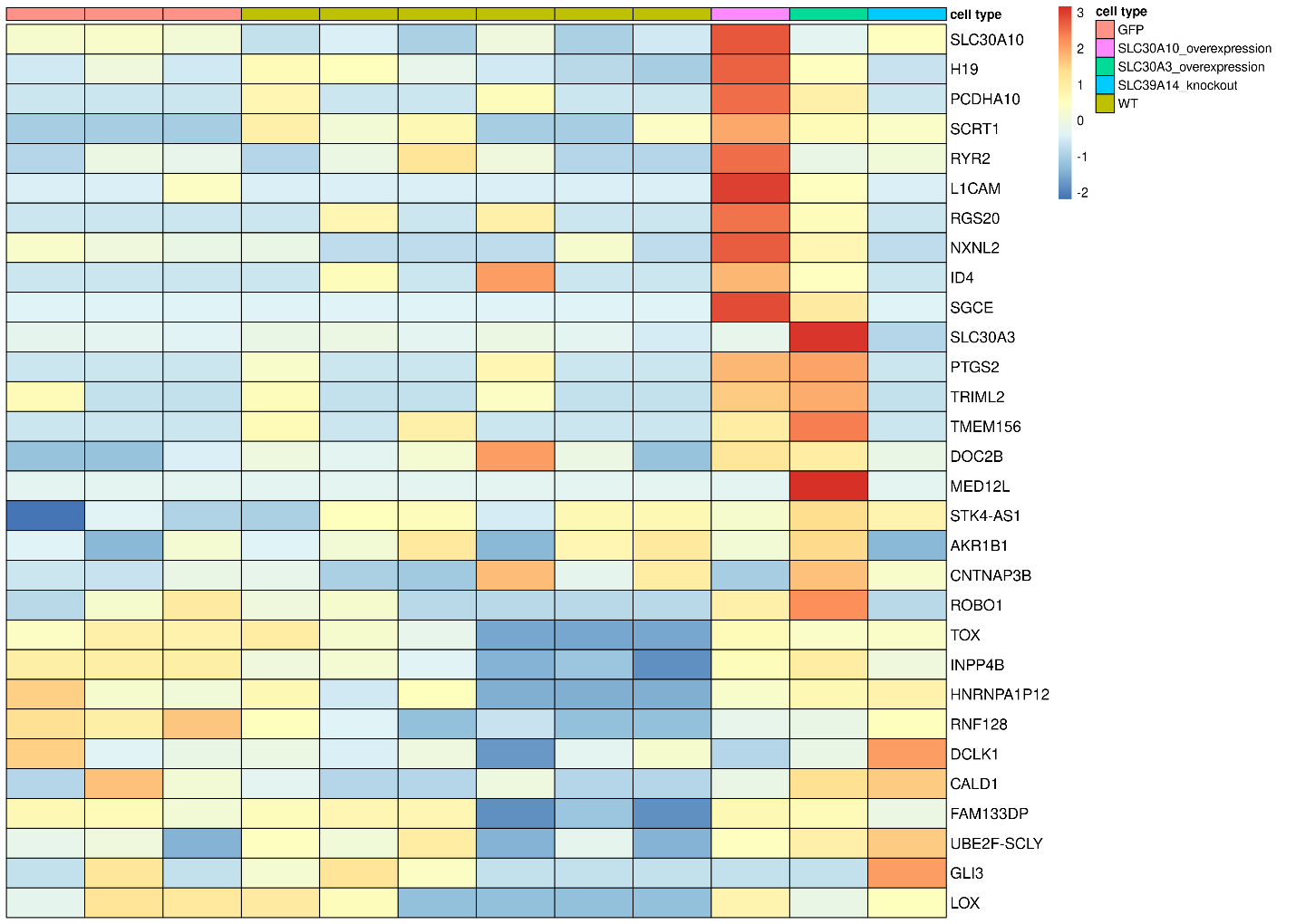


**Supplementary Figure S1. C) HCT-15 top upregulated genes.** From left to right, the figure displays control groups followed by the analyzed groups. Genes are arranged vertically in descending order of log2 fold change (log2FC), presenting the top 10 genes for each analyzed group, excluding controls. For the 'up' category, the genes represent the highest log2_FC values. The values within the cells correspond to z-scores.


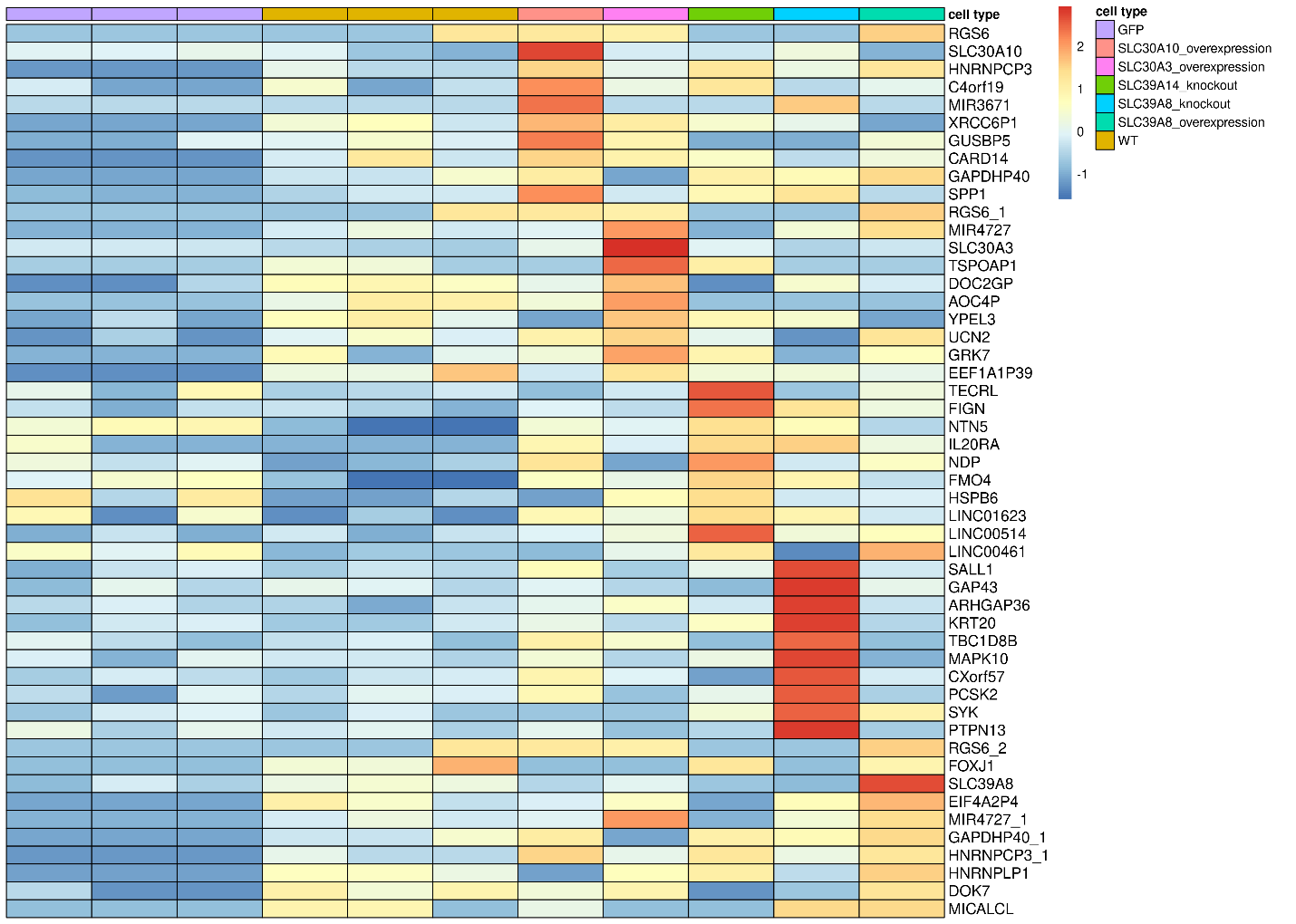


**Supplementary Figure S1. D) HuTu80 top upregulated genes.** From left to right, the figure displays control groups followed by the analyzed groups. Genes are arranged vertically in descending order of log2 fold change (log2FC), presenting the top 10 genes for each analyzed group, excluding controls. For the 'up' category, the genes represent the highest log2_FC values. The values within the cells correspond to z-scores.
